# Supplementary figures and images for: Predicting CD4 T-cell epitopes based on antigen cleavage, MHCII presentation, and TCR recognition
Source: PLoS One. 2018 Nov 6;13(11):e0206654. doi: 10.1371/journal.pone.0206654 (PMC6219782; doi:10.1371/journal.pone.0206654)

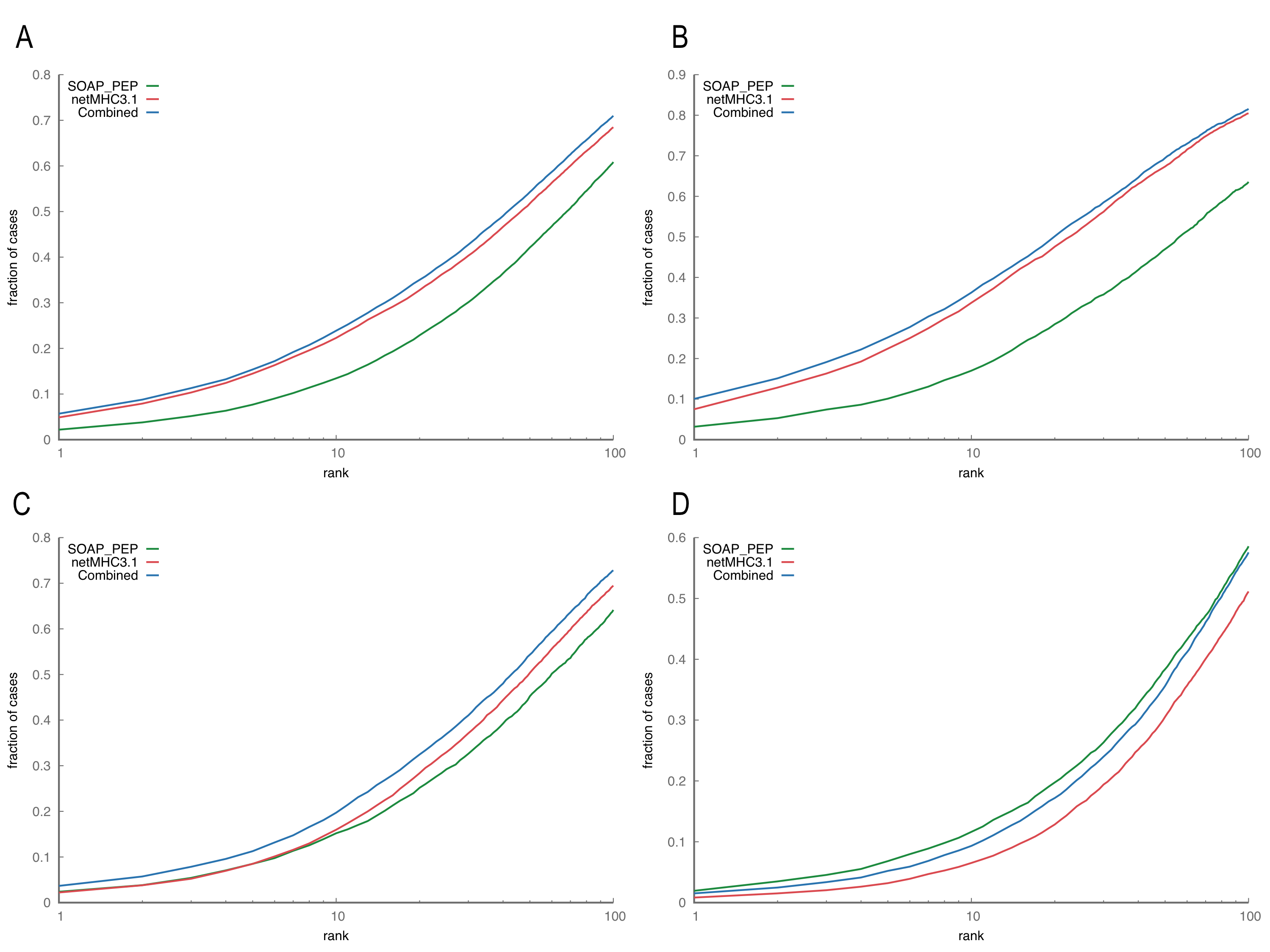

Supplement: S1 Fig — Success rate for SOAP_PEP, NetMHCIIpan3.1 and the two scoring functions combined for A) all, B) high, C) intermediate, and D) low affinity peptides. (TIFF) [file pone.0206654.s001.tiff]
