# Supplementary material for: Predicting CD4 T-cell epitopes based on antigen cleavage, MHCII presentation, and TCR recognition
Source: PLoS One. 2018 Nov 6;13(11):e0206654. doi: 10.1371/journal.pone.0206654 (PMC6219782; doi:10.1371/journal.pone.0206654)
Supplement: S1 Table — The predicted cleavage sites are marked with a dot. The confirmed epitope sequences from the ternary complex crystal structures are bolded. (DOCX) [file pone.0206654.s002.docx]

| **Protein** | **Number of 12-mer peptides before cleavage** | **Number of 12-mer peptides after cleavage** |
| --- | --- | --- |
| HEMAGGLUTININ HA1 | 306 | 159 |
| STATLC•LG•H•H•AVPNGTLVKTITDDQIEVTNATELVQ•SSSTGKICNNPHRILDGIDCTLIDALL•G•DPHCDVFQNETWDLFVERSKAFSNCYPYD  VPDYASLRSLVA•SSGTLEFITEGFT•W•TGVT•Q•NGGSNACK•RGPGSGFFSRLNWLT•KSGSTYPVLNVTMPNNDNFDKLY•IWG•IHHPSTNQE  QTSLYVQASGRVT•VSTRRSQQTIIPNIG•SRPWVR•GLSSRISIYWT•IVK•PGDVLVINSNGNLIAPRGYFKMRTGKSSIMR•SDAPIDTCISECITPN  GSIPNDKPFQNVNKITYGAC**PKYVKQNTLKLAT**GMRNVPE | | |
| TRIOSEPHOSPHATE ISOMERASE | 237 | 117 |
| APSRKFFVG•G•N•WK•M•N•GRKQSL**GELIGILNAAKVPAD**TEVVC•APPTAYIDFARQKLDPKIAVAAQNCYKVTNGAFTGEISPGMIKDCGATWV  VL•GHSERRHVFG•ESDELIGQKVAHALAE•GLGVIA•CIGEKLDEREAGITEKVVFEQTKVIADNVKDWSKVVLAYEPVW•AIGTGKTATPQQAQE  VHEKLRGWLK•SNVSDAVAQSTRIIYG•GSVT•GATCKELA•SQPDVDGFL•VG•GASLKPEFVDIINAKQ | | |
| MYELIN BASIC PROTEIN | 158 | 127 |
| AAQKRPSQRSKYLASASTMDHARHGFLPRHRDTGILDSLGRFFGSDRGAPKRGSGKDGHHAARTTHYGSLPQKAQGHRPQD**ENPVVHFFKN**  **IVT•PR**TPPPSQGKGRGLSLSR**FSWG•AEGQKPGFG•YGG**RASDYKSAHKGLKGHDAQGTLSKIFKLGGRDSRSGSPMARR | | |
| ENGA | 293 | 202 |
| ERRRRRGRRLDPQSLPIKLAIVGRPNVGKSTLTNRILGEERVVVYDMPGTTRDSIYIPMERDGREYVLID•TAGVRKRGKITDAVEKFSVIKTLQAI  EDANVVMLVIDAREGISDQDLSLLGFILNSGRSLVIVVNKWDGLSQEVKEQVKETLDFRLGFI**DFARVHFISALH•G•SG**VGNLFESVREAYDSSTR  RVGTSMLTRIM•T•MAVE•DHQPPLVRGRRVK•LKYA•H•AGGYNPPIVVIHGNQVK•DLPDSYKRYL•M•N•YFRKSLDVMGSPIRIQFKEGENPYANK  RNTLTPTQMR•KRKRLM•K•H•IKKNK | | |
| PHOSPHOMANNOMUTASE | 452 | 296 |
| MSTAKAPTLPASIFRAYDIRG•VVGDTLT•AETAYWIGRAIGSESLARGEPCVAVGRDGRLSGPELVKQLIQGLVDCGCQVSDVGMVPTPVLYYA  ANVLE•GKSGVM•LT•G•SHNPPDYNGFKIVVAGETLANEQIQALRERIEKNDLA•S•GVG•SVEQVDILPRYFK•QIRDDIAMAKPMK•VVVD•CGNG•V  A•G•VIAPQLIEALGCSVIPLYCEVDGNFPNHHPDPGKPENLKDLIAKVKAENADLGLA•FD•GDGDRVGVVT•NTGTIIYPD**RLLMLFAKDVVSRN**PG  ADIIFDVKCTRRLIALIS•GYGGRPVM•W•KTGHSLIKKKMK•ETGAL•LAGEMSGHVFFK•ERWFGFDDGIYSAARLLEILSQDQRDSEHVFSAFPSD  ISTPEINITVTEDSKFAIIEALQRDAQWGEGNITTLDGVRVDYPKGWGLVRASNTTPVLVLRFEADTEEELERIKTVFRNQLKAVDSSLPVPF | | |
| UL15 | 724 | 503 |
| M•FGQQLA•SDVQQYLERLEKQRQLKVGADEASAGLT•MGGDALRVPFLDFATATPKRHQTVVPGVG•TLHDCCEHSPLFSAVARRLLFNSLVPA  QLKGRDFGGDHTAKLEFLAPELVRAVARLRFKECAPADVVPQRNAYYSVLNTFQALHRSEAFR**QLVHFVRDFAQL**LKTSFRASSLT•ETTGPPKK  RAKVDVATHGRTYGTLELFQKMILM•H•ATYFLAAVL•L•G•DHAEQVNTFLRLVFEIPLFSDAAVRHFRQRATVFLVPRRHGKTWFLVPLIALSLASF  RGIKIGYTAHIRKATEPVFEEIDACLRG•W•FGSARVDHVK•GETISFSFPDGSRSTIVFASSHNTNGIRGQDFNLLFVDEANFIRPDAVQTIMGFLN•  QANCK•IIFVSSTNTGKASTSFLYNLR•GAADELLNVVTYICDDHMPRVVTHTNATACSCYILNKPVFITMDGAVRRTADLFLADSFMQEIIGGQARE  TGDDRPVLTKSAGERFLLYRPSTTTNSGLMAPDLYVYVDPAFTANTRASGTGVA•VVGRYRDDYIIFALEHFFLRA•LTG•SAPADIARCVVHSLTQ  VLA•LHPGAFRGVRVAVE•G•NSSQDSAVAIATHVHTEMHRLLASEGADA•G•SGPELLFYHCEPPGSAVLYPFFLLNKQKTPAFEHFIKKFNSGGV  M•ASQEIVSATVRLQ•TDPVEYLLEQLNNLT•ETVSPNTDVRTYSGKRNGASDDLMVAVIMAIYLA•A•QA•GPPHT•FAPITRVS | | |
| GLIADIN1 | 280 | 190 |
| MK•TFPILALLA•IVATTATTAVRVPVPQLQLQNPSQQQPQEQVPLVQEQQFQ•G•QQQPFPPQQPYPQPQPFPSQQPYLQL**QPFPQPELPYPQP**  QPFRPQQPYPQPQPQYSQPQQPISQQQQQQQQQQQQQQQILQ•QILQ•QQLIPCR•DVVL•QQHNIAHGSSQVLQESTYQLVQQLCCQQLWQI  PEQSRCQ•AIHNVVHAIILHQQH•H•H•H•QQQQQQQQQQPLSQVSFQ•QPQQQYPSGQGFFQPSQQNPQAQGSFQPQQLPQFEEIRNLALQTL  PAMCNVYIPPYCTIAPFG•IFG•TN | | |
| GLIADIN2 | 296 | 210 |
| MK•TFLILALLA•IVATTARIAVRVPVPQLQPQNPSQQQPQEQVPLVQ•QQQFPGQQQPFPPQQPYPQPQPFPSQQPYLQL**QPFPQPQLPYPQP**  QLPYPQPQLPYPQPQPFRPQQPYPQSQPQYSQPQQPISQQQQQQQQQQQQKQQQQQQQQILQ•QILQ•QQLIPCR•DVVL•QQHSIAYGSSQ  VLQ•QSTYQLVQQLCCQQLWQIPEQSRCQ•AIHNVVHAIILHQQQQQQQQQQQQPLSQVSFQ•QPQQQYP**SGEGSFQPSQENP**QAQGSVQP  QQLPQFEEIRNLALETLPAMCNVYIPPYCTIAPVG•IFG•TN | | |
| Invariant chain | 285 | 148 |
| MHRRRSRSCREDQKPVM•DDQRDLISNNEQLPMLGRRPGAPESKCSRGAL•YTGFSILVTLLLA•GQATTAYFLYQQQGRLDKLTVTSQNLQ•LE  NLRMKLPKPPK**PVSKMRMATPLLMQ**ALPMGALPQGPMQ•NATKYGNMT•EDHVM•HLL•Q•NADPLK•VYPPLKGSFPENLRHLKNTME•TIDWKV  F•E•S•W•M•H•H•WL•LFEMSRHSLE•QKPTDAPPKVLTKCQ•EEVSHIPAVHPGSFRPKCDENGNYLPLQCYG•SIGYCWCVFPNGTEVPNTRSRG  HHNCSESLELEDPSSGLGVTKQDLGPVPM | | |
