# Supplementary material for: Predicting CD4 T-cell epitopes based on antigen cleavage, MHCII presentation, and TCR recognition
Source: PLoS One. 2018 Nov 6;13(11):e0206654. doi: 10.1371/journal.pone.0206654 (PMC6219782; doi:10.1371/journal.pone.0206654)
Supplement: S2 Table — 1Alleles frequencies are taken from http://www.allelefrequencies.net. 2Highest sequence identity to a PDB structure. (DOCX) [file pone.0206654.s003.docx]

| HLA type | % in USA Caucasian Population^1^ | PDB code | Sequence identity^2^ |
| --- | --- | --- | --- |
| DRB1_0101 | 14.6 | 4e41 | 100% |
| DRB1_0102 | 3.9 | 3pdo | 99% |
| DRB1_0301 | 23.4 | 1a6a | 100% |
| DRB1_0401 | 17.3 | 2seb | 100% |
| DRB1_0402 | 1.9 | 2seb | 98% |
| DRB1_0404 | 6.1 | 2seb | 99% |
| DRB1_0405 | 0.7 | 2seb | 99% |
| DRB1_0407 | 2.1 | 2seb | 99% |
| DRB1_0701 | 23.4 | 3pdo | 90% |
| DRB1_0801 | 4.8 | 1j8h | 91% |
| DRB1_0802 | 0.8 | 3pdo | 91% |
| DRB1_1101 | 10.9 | 1ymm | 92% |
| DRB1_1104 | 4.9 | 1ymm | 92% |
| DRB1_1201 | 3.1 | 1ymm | 88% |
| DRB1_1301 | 10.0 | 1ymm | 92% |
| DRB1_1302 | 8.2 | 1a6a | 92% |
| DRB1_1401 | 4.8 | 1a6a | 93% |
| DRB1_1501 | 24.8 | 1ymm | 100% |
| DRB1_1601 | 4.7 | 1ymm | 97% |
| DRB4_0101 | 25.5 | 4ee41 | 87% |
| DRB4_0103 | 21 | 3pdo | 87% |
