# Supplementary material for: Predicting CD4 T-cell epitopes based on antigen cleavage, MHCII presentation, and TCR recognition
Source: PLoS One. 2018 Nov 6;13(11):e0206654. doi: 10.1371/journal.pone.0206654 (PMC6219782; doi:10.1371/journal.pone.0206654)
Supplement: S3 Table — The core residues that fit within the 9-residue MHCII groove are bolded. (DOCX) [file pone.0206654.s004.docx]

| **PDB** | **Protein** | **Confirmed MHCII binding peptide** | **Antigen length** | **MHCII type** | **SOAP Rank** | **NetMHCIIpan 3.1 Rank** | **Combined Rank** |
| --- | --- | --- | --- | --- | --- | --- | --- |
| 1a6a | CLIP GAMMA CHAIN | PVS**KMRMATPLLMQA** | 296 | DRB1_0301 | 58 | 2 | 2 |
| 1aqd | HLA CLASS I A-2 ALPHA CHAIN | GS**DWRFLRGYHQYA** | 365 | DRB1_0101 | 75 | 9 | 11 |
| 1fyt | HEMAGGLUTININ | **PKYVKQNTLKLA**T | 566 | DRB1_0101 | 21 | 11 | 10 |
| 1h15 | DNA POLYMERASE EPSTEIN-BARR VIRUS | **GGVYHFVKKHVH**ES | 1015 | DRB5_0101 | 530 | 53 | 67 |
| 1j8h | HEMAGGLUTININ | **PKYVKQNTLKLA**T | 566 | DRB1_0401 | 12 | 1 | 3 |
| 1jk8 | INSULIN | **LVEALYLVCGER**GG | 110 | DQA10301-DQB10302 | 2 | 2 | 1 |
| 1sjh | GAG POLYPROTEIN HIV | **PEVIPMFSALSE**G | 501 | DRB1_0101 | 121 | 14 | 24 |
| 1uvq | OREXIN | **MNLPSTKVSWAA**V | 131 | DQA10602-DQB10602 | 1 | 61 | 3 |
| 1ymm | MYELIN BASIC PROTEIN | EN**PVVHFFKNIVTP** | 304 | DRB1_1501 | 6 | 2 | 1 |
| 1zgl | MYELIN BASIC PROTEIN | V**HFFKNIVTPRTP**G | 304 | DRB5_0101 | 9 | 2 | 1 |
| 2fse | COLLAGEN ALPHA-1(II) | **AGFKGEQGPKGE**PG | 1487 | DRB1_0101 | 286 | 147 | 126 |
| 2iam | TRIOSEPHOSPHATE ISOMERASE | G**ELIGILNAAKVP**AD | 286 | DRB1_0101 | 1 | 1 | 1 |
| 2ian | TRIOSEPHOSPHATE ISOMERASE | G**ELIGTLNAAKVP**AD | 286 | DRB1_0101 | 1 | 3 | 1 |
| 2nna | GLIADIN | **SGEGSFQPSQEN**P | 307 | DQA10301-DQB10302 | 1 | 41 | 1 |
| 2q6w | PLATELET INTEGRIN | **AWRSDEALPLG** | 788 | DRB3_0101 | 129 | 9 | 5 |
| 2seb | COLLAGEN II | **QYMRADQAAGGL** | 1487 | DRB1_0401 | 571 | 8 | 13 |
| 2wbj | ENGA | DF**ARVHFISALHGS**G | 304 | DRB1_1501 | 181 | 1 | 1 |
| 3c5j | ELONGATION FACTOR 1-ALPHA 2 | **QVIILNHPGQIS**A | 463 | DRB3_0101 | 102 | 15 | 14 |
| 3l6f | MART-1 | AP**PAYEKLSAEQSP**P | 118 | DRB1_0101 | 23 | 5 | 4 |
| 3lqz | HUMAN HLA CLASS DR ALPHA | II**RKFHYLPFLPST** | 254 | DPA10103-DPB10201 | 1 | 2 | 1 |
| 3o6f | MYELIN BASIC PROTEIN | **FSWGAEGQRPGF**G | 304 | DRB1_0401 | 152 | 104 | 138 |
| 3pdo | CLIP GAMMA CHAIN | PVS**KMRMATPLLMQA**LP | 296 | DRB1_0101 | 116 | 2 | 4 |
| 3pl6 | MYELIN BASIC PROTEIN | N**PVVHFFKNIVTP**R | 304 | DQA10102-DQB10502 | 2 | 1 | 1 |
| 4d8p | GLIADIN | P**QPQQPQQPFPQP** | 285 | DQA10301-DQB10201 | 151 | 263 | 255 |
| 4grl | PHOSPHOMANNOMUTASE | R**LLMLFAKDVVSR**N | 463 | DQA10102-DQB10502 | 22 | 19 | 9 |
| 4is6 | MELANOCYTE PROTEIN PMEL | **RQLYPEWTEAQR**L | 661 | DRB1_0401 | 118 | 168 | 135 |
| 4may | UL15 | **QLVHFVRDFAQL** | 735 | DQA10102-DQB10502 | 3 | 10 | 2 |
| 4ov5 | HLA CLASS I A-2 ALPHA CHAIN | G**SDARFLRGYHLY**A | 365 | DRB1_0101 | 66 | 9 | 10 |
| 4ozh | GLIADIN | **PFPQPELPYPQP**Q | 291 | DQA10501-DQB10201 | 60 | 226 | 177 |
| 4ozi | GLIADIN | **QPFPQPELPYP** | 291 | DQA10501-DQB10201 | 13 | 143 | 84 |
| 4p5m | MHC CLASS I ANTIGEN HLA-A28 | **QAYDGKDYIALK**G | 273 | DPA10103-DPB10201 | 20 | 67 | 49 |
| 4y19 | INSULIN | **QPLALEGSLQKR**G | 110 | DRB1_0401 | 8 | 9 | 8 |
| Number of cases out of total with the correct MHCII binding peptide ranked #1 | | | | | 5 | 4 | 9 |
