# Supplementary material for: Predicting CD4 T-cell epitopes based on antigen cleavage, MHCII presentation, and TCR recognition
Source: PLoS One. 2018 Nov 6;13(11):e0206654. doi: 10.1371/journal.pone.0206654 (PMC6219782; doi:10.1371/journal.pone.0206654)
Supplement: S4 Table — RMSD to closest structure in the template library in the last column. (DOCX) [file pone.0206654.s005.docx]

|  | 1j8h | 1fyt | 4e41 | 2iam | 2ian | 1ymm | 2wbj | 3pl6 | 4grl | 4may | 3o6f | 1zgl | 4ozf | 4ozg | 4ozh | 4ozi | 4gg6 | 4z7u | 4z7v | 4z7w |  |
| --- | --- | --- | --- | --- | --- | --- | --- | --- | --- | --- | --- | --- | --- | --- | --- | --- | --- | --- | --- | --- | --- |
| 1j8h | 0.0 | **0.4** | 9.3 | 8.2 | 9.0 | 17.3 | 16.5 | 7.2 | 7.3 | 7.1 | 4.4 | 6.5 | 8.2 | 5.1 | 4.2 | 10.0 | 7.1 | 4.1 | 6.2 | 5.2 | 1.5 |
| 1fyt | **0.4** | 0.0 | 9.6 | 8.4 | 9.2 | 17.6 | 16.6 | 7.1 | 7.2 | 6.9 | 4.4 | 6.7 | 8.4 | 5.3 | 4.4 | 10.4 | 6.6 | 4.4 | 6.3 | 6.2 | 1.6 |
| 4e41 | 9.3 | 9.6 | 0.0 | 3.1 | **2.9** | 12.8 | 10.6 | 15.8 | 16.0 | 15.3 | 11.9 | 5.9 | 9.8 | 7.3 | 7.8 | 9.0 | 8.5 | 7.6 | 7.0 | 6.3 | 1.0 |
| 2iam | 8.2 | 8.4 | 3.1 | 0.0 | **0.9** | 14.1 | 12.9 | 13.9 | 14.1 | 13.4 | 9.7 | 3.4 | 9.4 | 5.1 | 5.3 | 8.5 | 7.5 | 5.8 | 6.7 | 6.1 | 1.7 |
| 2ian | 9.0 | 9.2 | 2.9 | **0.9** | 0.0 | 14.8 | 13.0 | 14.4 | 14.6 | 14.0 | 10.4 | 4.7 | 10.0 | 5.7 | 5.9 | 8.9 | 8.0 | 6.4 | 7.2 | 6.8 | 1.5 |
| 1ymm | 17.3 | 17.6 | 12.8 | 14.1 | 14.8 | 0.0 | **2.2** | 23.9 | 24.0 | 23.9 | 20.3 | 14.0 | 20.5 | 18.3 | 18.1 | 19.5 | 20.7 | 18.6 | 18.0 | 15.4 | 1.5 |
| 2wbj | 16.5 | 16.6 | 10.6 | 12.9 | 13.0 | **2.2** | 0.0 | 21.7 | 22.0 | 21.7 | 19.5 | 12.4 | 19.4 | 16.8 | 15.7 | 18.4 | 18.7 | 16.8 | 17.4 | 13.4 | 1.2 |
| 3pl6 | 7.3 | 7.1 | 15.8 | 13.9 | 14.4 | 23.9 | 21.7 | 0.0 | 0.5 | **0.4** | 5.0 | 12.2 | 8.6 | 8.8 | 8.3 | 11.4 | 10.7 | 8.6 | 9.4 | 11.1 | 1.4 |
| 4grl | 7.4 | 7.2 | 16.0 | 14.1 | 14.6 | 24.0 | 22.0 | **0.5** | 0.0 | 0.8 | 5.5 | 12.4 | 8.2 | 9.1 | 8.6 | 11.3 | 11.1 | 8.9 | 11.7 | 11.2 | 1.4 |
| 4may | 7.1 | 6.9 | 15.3 | 13.4 | 14.0 | 23.9 | 21.7 | **0.4** | 0.8 | 0.0 | 4.2 | 12.1 | 8.2 | 8.7 | 8.1 | 12.3 | 10.6 | 8.3 | 11.2 | 11.1 | 1.4 |
| 3o6f | 4.4 | 4.4 | 11.9 | 9.7 | 10.4 | 20.3 | 19.5 | 5.0 | 5.5 | **4.2** | 0.0 | 8.7 | 6.9 | 5.4 | 5.2 | 9.3 | 5.2 | 5.1 | 5.4 | 8.8 | 2.1 |
| 1zgl | 6.5 | 6.7 | 5.9 | **3.4** | 4.7 | 14.0 | 12.4 | 12.2 | 12.4 | 12.1 | 8.7 | 0.0 | 10.0 | 5.3 | 5.0 | 10.5 | 7.3 | 6.2 | 6.8 | 6.3 | 2.9 |
| 4ozf | 8.2 | 8.4 | 9.8 | 9.4 | 10.0 | 20.5 | 19.4 | 8.6 | 8.2 | 8.2 | 6.9 | 10.0 | 0.0 | 5.4 | 5.5 | 4.0 | 4.2 | 4.6 | **3.9** | 5.5 | 2.1 |
| 4ozg | 5.1 | 5.3 | 7.3 | 5.1 | 5.7 | 18.3 | 16.8 | 8.8 | 9.1 | 8.7 | 5.4 | 5.3 | 5.4 | 0.0 | **0.5** | 6.4 | 3.4 | 1.8 | 2.5 | 4.6 | 2.0 |
| 4ozh | 4.2 | 4.4 | 7.8 | 5.3 | 5.9 | 18.1 | 15.7 | 8.3 | 8.6 | 8.1 | 5.2 | 5.0 | 5.5 | **0.5** | 0.0 | 6.6 | 5.2 | 1.9 | 2.5 | 4.8 | 1.4 |
| 4ozi | 10.0 | 10.4 | 9.0 | 8.5 | 8.9 | 19.5 | 18.4 | 11.4 | 11.3 | 12.3 | 9.3 | 10.5 | **4.0** | 6.4 | 6.6 | 0.0 | 6.4 | 6.3 | 6.0 | 5.8 | 4.2 |
| 4gg6 | 7.1 | 6.6 | 8.5 | 7.5 | 8.0 | 20.7 | 18.7 | 10.7 | 11.1 | 10.6 | 5.2 | 7.3 | 4.2 | 3.4 | 5.2 | 6.4 | 0.0 | **1.5** | 1.8 | 6.6 | 1.4 |
| 4z7u | 4.1 | 4.4 | 7.6 | 5.8 | 6.4 | 18.6 | 16.8 | 8.6 | 8.9 | 8.3 | 5.1 | 6.2 | 4.6 | 1.8 | 1.9 | 6.3 | 1.5 | 0.0 | **1.4** | 4.2 | 1.0 |
| 4z7v | 6.2 | 6.3 | 7.0 | 6.7 | 7.2 | 18.0 | 17.4 | 9.4 | 11.7 | 11.2 | 5.4 | 6.8 | 3.9 | 2.5 | 2.5 | 6.0 | 1.8 | **1.4** | 0.0 | 3.5 | 1.4 |
| 4z7w | 5.2 | 6.2 | 6.3 | 6.1 | 6.8 | 15.4 | 13.4 | 11.1 | 11.2 | 11.1 | 8.8 | 6.3 | 5.5 | 4.6 | 4.8 | 5.8 | 6.6 | 4.2 | **3.5** | 0.0 | 2.2 |
