# Supplementary material for: Predicting CD4 T-cell epitopes based on antigen cleavage, MHCII presentation, and TCR recognition
Source: PLoS One. 2018 Nov 6;13(11):e0206654. doi: 10.1371/journal.pone.0206654 (PMC6219782; doi:10.1371/journal.pone.0206654)
Supplement: S5 Table — (DOCX) [file pone.0206654.s006.docx]

1ao7 1bd2 1bwm 1d9k 1fyt 1h5b 1j8h 1jck 1kgc 1ktk 1l0y 1lp9 1mi5 1oga 1qrn 1qse 1qsf 1s8d 1t1w 1t1x 1t1y 1t1z 1t20 1t21 1t22 1tcr 1tvd 1u3h 1uqs 1uxs 1uxw 1w72 1ymm 1zgl 1zsd 2ak4 2av1 2av7 2axh 2axj 2bnq 2bnr 2bnu 2bsr 2bss 2bst 2bvo 2bvp 2bvq 2c7u 2cde 2cdf 2cdg 2cii 2cik 2e7l 2esv 2f53 2gj6 2hjk 2hjl 2ial 2iam 2ian 2icw 2ij0 2j8u 2jcc 2nw2 2nx5 2oi9 2ozo 2p1y 2p5e 2p5w 2pxy 2pye 2pyf 2q86 2uwe 2v2w 2v2x 2vlj 2vlk 2vll 2vlm 2vlr 2wbj 2x4n 2x4o 2x4p 2x4q 2x4r 2x4s 2x4t 2x4u 2x70 2x89 2xn9 2xna 2xpg 2ypl 2z31 2z35 3arb 3ard 3are 3arf 3arg 3axl 3bo8 3bp6 3c60 3c6l 3d25 3d39 3d3v 3dx9 3dxa 3ffc 3fqn 3fqr 3fqt 3fqu 3fqw 3fqx 3gsn 3gso 3gsq 3gsr 3gsu 3gsv 3gsw 3gsx 3h7b 3h9h 3h9s 3he6 3he7 3ik5 3ioz 3ixa 3kla 3kpl 3kpm 3kpn 3kpo 3kpp 3kpq 3kpr 3kps 3kww 3kxf 3mff 3mfg 3mr9 3mrb 3mrc 3mrd 3mre 3mrf 3mrg 3mrh 3mri 3mrj 3mrk 3mrl 3mrm 3mrn 3mro 3mrp 3mrq 3mrr 3mv7 3mv8 3mv9 3nfn 3o4l 3o6f 3o8x 3o9w 3od6 3ody 3odz 3oef 3of6 3ox8 3oxr 3oxs 3pl6 3pqy 3pwj 3pwl 3pwn 3pwp 3q5t 3qdg 3qdj 3qdm 3qeq 3qeu 3qfd 3qfj 3qh3 3qi9 3qib 3qjf 3qux 3quy 3r8b 3rdt 3rev 3ri4 3rtq 3rug 3rzc 3scm 3sda 3sdc 3sdd 3sdx 3sjv 3skm 3skn 3sko 3spv 3t0e 3ta3 3tfk 3tjh 3tn0 3tpu 3tvm 3tzv 3u0p 3utp 3utq 3uts 3utt 3vcl 3vfm 3vfn 3vfo 3vfp 3vfr 3vfs 3vft 3vfu 3vfv 3vfw 3vwj 3vwk 3vxm 3vxn 3vxo 3vxp 3vxq 3vxr 3vxs 3vxt 3vxu 3w0w 4aen 4ah2 4dzb 4ei5 4ei6 4elk 4en3 4eup 4euq 4eur 4ftv 4g8e 4g8f 4g8g 4g8i 4g9d 4g9f 4gkn 4gks 4gkz 4grl 4grm 4h1l 4h25 4h26 4i4w 4iiq 4irj 4irs 4jfd 4jfe 4jff 4jfh 4jfo 4jfp 4jfq 4jrx 4jry 4k7f 4l4t 4l4v 4l8s 4l9l 4lcc 4lcw 4lfh 4lhu 4may 4mng 4mnh 4mnq 4mq7 4ndm
